# Changes in urinary renal injury markers in children with *Mycoplasma pneumoniae* pneumonia and a prediction model for related early renal injury

**DOI:** 10.1186/s13052-024-01709-7

**Published:** 2024-08-23

**Authors:** Ju Zhang, He-kai Ma, Bao-wen Li, Ke-Ke Ma, Yu-Ling Zhang, Shu-jun Li

**Affiliations:** Department of Pediatrics, The First Affiliated Hospital of Xinxiang Medical University No. 88, Weihui Jianjian Road, Xinxiang, 453100 China

**Keywords:** Kidney injury, Biomarkers, *Mycoplasma pneumoniae* pneumonia, Children

## Abstract

**Background:**

This study aims to analyse changes in urinary kidney injury markers in children with *Mycoplasma pneumoniae* pneumonia (MPP), investigate the risk factors for MPP-related acute kidney injury (AKI) and establish a model to predict MPP-related AKI.

**Methods:**

Ninety-five children were enrolled based on the study’s inclusion and exclusion criteria. They were divided into a severe MPP (SMPP) group and a non-SMPP group and then into an AKI group and a non-AKI group according to the presence of AKI. A univariate logistic regression analysis was performed to explore the early risk factors for AKI. Based on a multivariate logistic regression analysis and a least absolute shrinkage and selection operator regression analysis, appropriate variables were selected to establish a prediction model, and R 4.2.2 software was used to draw nomograms and generate a dynamic nomogram website.

**Results:**

Seven urinary kidney injury markers were abnormally elevated in the SMPP group and the non-SMPP group: urinary N-acetyl-β-D-glucosaminidase (NAG), β2-microglobulin, α1-microglobulin, retinol-binding protein, urinary immunoglobulin G, urinary transferrin and urinary microalbumin. Sixteen children were identified with AKI during hospitalisation. The AKI group had higher levels of urinary NAG, α1-microglobulin, β2-microglobulin, urinary microalbumin, urinary transferrin and retinol-binding protein than the non-AKI group (*P* < 0.05). The MPP-related AKI prediction model consists of four indicators (serum immunoglobulin M [IgM], C-reactive protein [CRP], urine NAG and sputum plug presence) and a dynamic nomogram.

**Conclusion:**

Urinary kidney injury markers are often elevated in children with MPP; urinary NAG is the marker most likely to be elevated, and it is especially evident in severe cases. The nomogram of the prediction model, comprising serum IgM, CRP, urinary NAG and sputum plug presence, can predict the probability of AKI in children with MPP.

**Supplementary Information:**

The online version contains supplementary material available at 10.1186/s13052-024-01709-7.

## Introduction

The incidence of *Mycoplasma pneumoniae* pneumonia (MPP) in children worldwide is increasing annually. Paediatric MPP is mostly mild and self-limited, but it may develop into severe MPP (SMPP) or refractory MPP (RMPP), which often require hospitalisation. Acute kidney injury (AKI) is a sudden decline in renal function resulting from a variety of causes; AKI occurs in approximately 5% of hospitalised patients and in more than 30% of patients in intensive care units (ICUs) [1, 2]. Our preliminary research found that some children with SMPP admitted to ICUs had AKI during the course of the disease; however, if identified early and treated in a stable environment with nutritional support and the prevention or treatment of complications, the prognosis improved.

Recently, many urinary biomarkers have been identified, but there are few studies on their changes in children with MPP and whether they can be used to predict the occurrence of MPP-related AKI. Therefore, this study statistically analysed the changes in urinary kidney injury markers in children with MPP, explored the possible risk factors for MPP-related AKI and established a model to predict MPP-related AKI to guide the clinical early detection of MPP-related kidney injury and the provision of early interventions to prevent AKI from progressing to renal failure and chronic kidney disease.

## Materials and methods

### Research participants

**Fig. 1:**
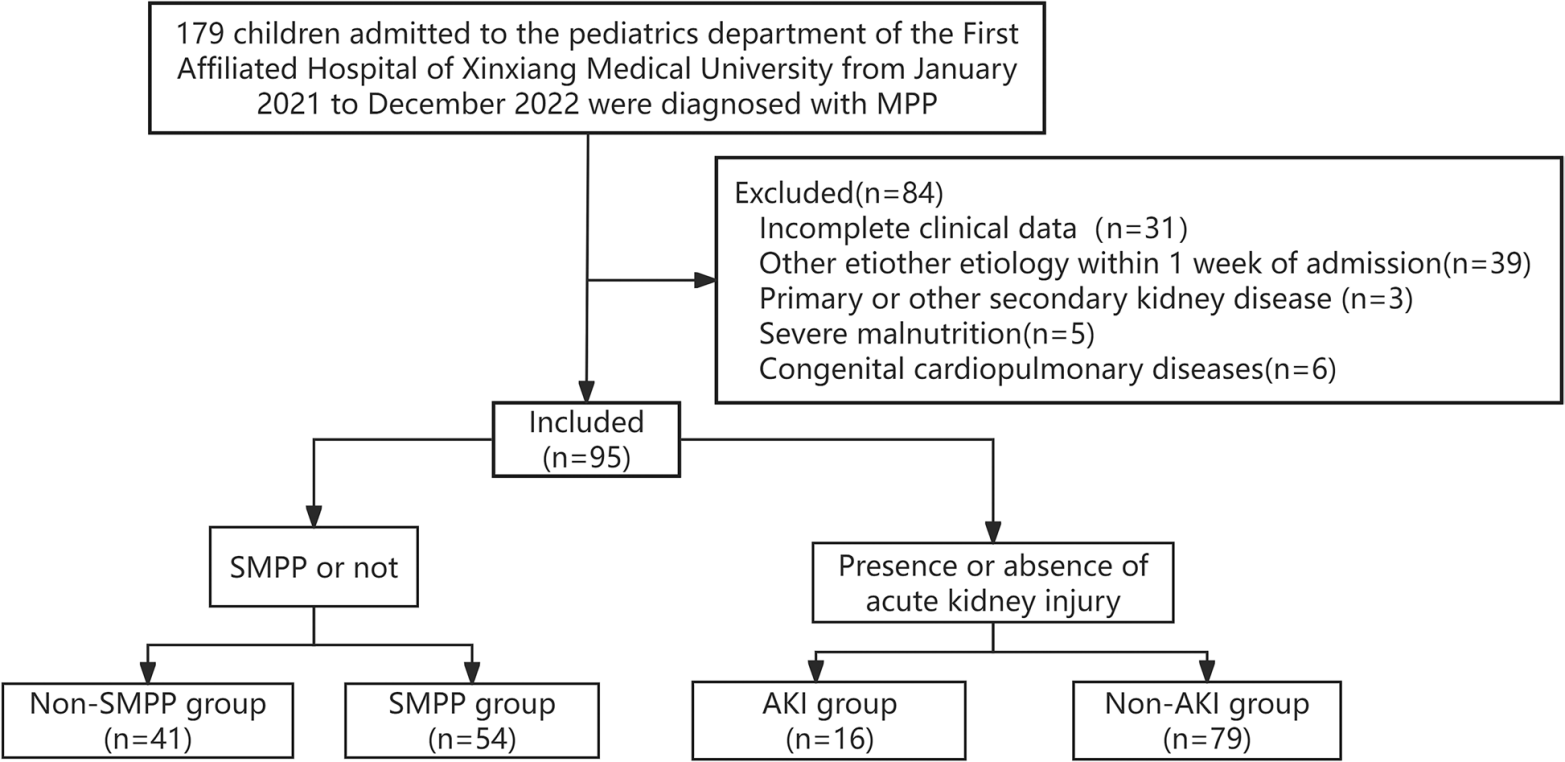
Flow chart of patient inclusion

This was a prospective study. A total of 179 children admitted to the paediatrics department of the First Affiliated Hospital of Xinxiang Medical University between January 2021 and December 2022 were diagnosed with MPP. Of these, 84 were excluded from the study and 95 (50 boys and 45 girls) were divided into an SMPP group and a non-SMPP group. The children with AKI were defined as the AKI group, and the remaining children were defined as the non-AKI group (Fig. 1). All children with AKI were in stage 1 of the condition. The study was approved by the scientific research ethics committee of the First Affiliated Hospital of Xinxiang Medical College (No. 2020029), and the guardians of all the enrolled children gave their signed informed consent.

### Inclusion and exclusion criteria

The diagnosis of MPP, SMPP and RMPP corresponded to the diagnostic criteria of the *Expert Consensus on the Diagnosis and Treatment of Mycoplasma Pneumoniae Pneumonia in Children* (2015 edition) [3].

Diagnosis of MPP: In accordance with the typical clinical and imaging manifestations of MPP, the disease was diagnosed if at least one of the following was detected: 1) single serum MP antibody titre ≥ 1:160 (PA method), double serum MP during the course of the disease and antibody titres that had at least quadrupled; 2) MP-DNA or RNA positive.

Diagnosis of SMPP: A diagnosis was made if any one of the following symptoms was present: persistent high fever (above 39 °C) for ≥ 5 days or fever for ≥ 7 days, with no downward trend in peak body temperature; wheezing, shortness of breath, dyspnoea, chest pain or haemoptysis. Intrapulmonary complications, such as moderate-to-large pleural effusion, large-scale lung consolidation and necrosis, and pulmonary embolism; extrapulmonary complications, such as those related to the skin and mucous membranes, nervous system, blood system, circulatory system, kidney, liver and pancreas; finger pulse oxygen saturation ≤ 0.93 when breathing air in a calm state; imaging indicating one of the following conditions: 1) ≥ 2/3 of a single lung lobe affected, uniform high-density consolidation or high-density consolidation appearing in two or more pulmonary lobes (consolidation, regardless of the size of the involved area, could be accompanied by moderate-to-large pleural effusion or localised bronchiolitis); 2) diffuse bronchiolitis in one lung or ≥ 4/5 of the bilateral lobes, possibly complicated by bronchitis and mucus plug formation, leading to atelectasis.

Diagnostic criteria for AKI: An increase in serum creatinine by ≥ 0.3 mg/dl (≥ 26.5 µmol/l) within 48 h; an increase in serum creatinine to ≥ 1.5 times baseline within the previous 7 days; urine volume ≤ 0.5 ml/kg/h for 6 h, except for urinary obstruction or other reversible factors, causing a reduction in urine volume. The above three criteria satisfied any one diagnostic. Stage 1 of AKI: serum creatinine increased 1.5–1.9 times from baseline or was > 26.5 µmol/l; urine volume < 0.5 ml / (kg·h) for 6–12 h [4].

Exclusion criteria: (1) incomplete clinical data; (2) other aetiological infections within 1 week of admission; (3) primary or other secondary kidney diseases (haemolytic anaemia, allergic purpura, rhabdomyolysis, etc.); (4) severe malnutrition; (5) congenital cardiopulmonary diseases.

### Methods

The general information, clinical characteristics, diagnosis and treatment processes, and laboratory test results of the enrolled children were collected individually for statistical analysis and comparisons between the groups. The treatment of MPP was based on the *Expert Consensus on the Diagnosis and Treatment of Mycoplasma Pneumoniae Pneumonia in Children* (2015 edition). Standardised diagnosis and treatment were performed in combination with the clinical presentation of the children, and the timing of the application of various drugs and treatment methods was strictly controlled. In accordance with the Chinese guidelines on paediatric flexible bronchoscopy (2018 edition) [5], the indications for electronic bronchoscopy and the treatment of the enrolled children were strictly controlled. Routine bronchoscopy and treatment are not recommended for mild cases. In children with SMPP and suspected mucus plug blockage, treatment should proceed as early as possible to reduce the occurrence of complications and sequelae. Comprehensive analysis was conducted based on clinical manifestations, physical signs and laboratory test results to determine the presence of extrapulmonary complications. These complications included myocardial damage, liver function damage, coagulation disorders, pericardial effusion, meningitis and AKI.

On the day of admission, the families of all the enrolled children signed a consent form for the examination. The nurse in charge collected blood samples in a standardised manner and sent them to the relevant laboratory of our hospital for examination. Morning urine samples were also collected and sent to the hospital’s laboratory for urinary and renal examination. The joint detection of injury markers, including α1-microglobulin, β2-microglobulin, urinary immunoglobulin G (IgG), retinol-binding protein, urinary transferrin, urinary microalbumin and urinary N-acetyl-β-D-glucosaminidase (NAG), was performed.

### Statistical analysis

For the statistical processing of data, SPSS 26.0, GraphPad Prism 8 and R 4.2.2 software were used. Measurement data were tested for normality using the D’Agostino test. Measurement data that conformed to a normal distribution were expressed as mean ± standard deviation, and an independent-samples *t*-test was used for comparisons between groups; those that did not conform to a normal distribution were expressed as medians. Numbers and interquartile ranges were expressed as M (P25, P75), and non-parametric tests (Mann–Whitney U test) were used for comparisons between groups. Count data were expressed as rates (*n* [%]), and comparisons between groups were conducted using the Chi-squared test or Fisher’s exact test. A *P*-value < 0.05 was considered to represent a statistically significant difference. The receiver operating characteristic (ROC) curve was used to analyse the diagnostic value of the urinary kidney injury markers and urinary NAG for AKI.

#### Construction of the prediction model

A single-factor logistic regression analysis was used to explore the early risk factors for AKI. Based on these results, the stepwise regression method was used to conduct a multi-factor logistic regression analysis for variables with* P* < 0.01 and to screen for meaningful variables. These variables were incorporated into the AKI early prediction model. The bootstrap verification method was used to verify the diagnostic efficiency of the model. Based on the prediction model, R software was used to draw a nomogram and generate a dynamic nomogram website.

## Results

### Baseline data for the SMPP and non-SMPP groups

The enrolled patients ranged in age from 1 month to 16 years, with a median age of 7 years. No significant differences between the two groups were identified in terms of gender, peak fever temperature, cough, wheezing, bronchoscopic sputum plugs, white blood cell count, platelet count or interleukin-6 levels (*P* > 0.05). However, the SMPP group had a higher percentage of patients experiencing dyspnoea than the non-SMPP group (*P* < 0.05) as well as a longer fever duration (*P* < 0.05). The non-SMPP group exhibited higher levels of MP-DNA copy numbers, lactate dehydrogenase, serum IgG and immunoglobulin M (IgM), as well as lower neutrophil ratios and C-reactive protein (CRP), alanine transaminase, aspartate aminotransferase, creatine kinase and D-dimer levels, all with significant differences (*P* < 0.05). Additionally, the SMPP group had higher serum creatinine levels, and the glomerular filtration rate and urine output were lower in the SMPP group than in the non-SMPP group (*P* < 0.05), as shown in Table 1.

**Table 1:** Comparison of clinical data and other laboratory examination results in the SMPP group and non-SMPP group

	SMPP group (*n* = 54)	Non-SMPP group (*n* = 41)	*P value*
Gender			0.307
Male	31(57.4%)	19(46.3%)	
Female	23(42.6%)	22(53.7%)	
Age(y)	6.55 ± 3.08	7.02 ± 2.80	0.450
Cough, n(%)	52(96.3%)	41(100%)	0.504
Wheezing, n(%)	7(13.0%)	5(12.2%)	1
Sputum plugs, n(%)	23(42.6%)	13(31.7%)	0.192
Dyspnea, n(%)	16(29.6%)	0	** < 0.001**
Length of hospitalization (d)	15(10 ~ 20)	11(8 ~ 13)	**0.001**
Fever peaks (℃)	39.55 ± 0.75	39.33 ± 0.65	0.169
Days with feve (d)	12(8.25 ~ 17.50)	10.5(6.0 ~ 13.25)	**0.041**
MP-DNA (copies/mL)	2.65 × 10^7^(1.56 × 10^5^ ~ 2.10 × 10^8^)	3.66 × 10^6^(2.52 × 10^4^ ~ 3.67 × 10^7^)	**0.048**
WBC (× 10^9^/L)	9.57 ± 4.13	9.17 ± 5.13	0.701
NE%	73.54 ± 13.80	61.67 ± 16.47	**0.001**
PLT (× 10^9^/L)	310(229.25 ~ 382.25)	316.5(273.25 ~ 433.25)	0.196
CRP (mg/L)	35.15(8.20 ~ 100.45)	9.72(2.94 ~ 39.03)	**0.014**
IL-6 (pg/mL)	22.59(7.04 ~ 50.31)	15.52(7.46 ~ 30.76)	0.210
ALT(U/L)	36.5(19.25 ~ 67.5)	19.5(13.75 ~ 36.25)	**0.014**
AST(U/L)	43(26.75 ~ 57.75)	29(21 ~ 33)	< 0.001
LDH(U/L)	53(36.25 ~ 125)	303.5(241.75 ~ 437.5)	** < 0.001**
CK(U/L)	538.5(268.25 ~ 1043)	62.5(33.25 ~ 105.75)	** < 0.001**
D-Dimer(ug/mL)	3.05(1.23 ~ 8.18)	1.55(0.98 ~ 3.00)	**0.028**
Serum IgG	7.95(5.9 ~ 9.8)	9.2(7.9 ~ 12)	0.005
Serum IgM	1.36(0.94 ~ 2.33)	1.93(1.45 ~ 3.39)	0.002
GFR(ml/min/1.73m^2^)	101.16(94.85 ~ 107.46)	129.95(124.29 ~ 135.62)	** < 0.001**
Serum creatinine(umol/L)	48.47(43.28 ~ 53.66)	35.09(32.52 ~ 37.66)	** < 0.001**
Urine output(ml/24h)	605.74(538.58 ~ 772.91)	829.27(777.47 ~ 881.07)	** < 0.001**

*WBC* White Blood Cell Count, *NE%* Neutrophil Percentage, *PLT* Platelet Count, *CRP* C-Reactive Protein, *IL-6* Interleukin-6, *ALT* Alanine Aminotransferase, *LDH* Lactate Dehydrogenase, *CK* Creatine Kinase

### Comparison of urinary kidney injury markers in the SMPP and non-SMPP groups

The seven urinary kidney injury markers exhibited abnormal increases in the SMPP group. The marker most likely to increase was urinary NAG (68.42%), followed by β2-microglobulin (53.68%), α1-microglobulin (28.42%), retinol-binding protein (22.11%), urinary IgG (12.63%), urinary transferrin (9.47%) and urinary microalbumin (3.16%) (see Supplementary Table 1). The urinary microalbumin and urinary IgG levels of the two groups were not significantly different (*P* > 0.05); however, the levels of α1-microglobulin, β2-microglobulin, retinol-binding protein, urinary transferrin and urinary NAG were significantly higher in the SMPP group, with statistically significant differences (*P* < 0.05) (see Table 2).

**Table 2:** Comparison of urinary kidney injury markers and urinary NAG values between non-SMPP and SMPP groups

	Non-SMPP group (*n* = 41)	SMPP group (*n* = 54)	*Z* value	*P* value
α1 microglobular protein(U/L)	5.3(2.05,10.8)	8.65(4.93,17.5)	-2.773	0.006
β2 microglobulin(U/L)	0.3(0.14,0.48)	0.41(0.21,1.75)	-2.266	0.023
Retinol binding protein(U/L)	0.13(0.08,0.29)	0.32(0.12,0.94)	-2.537	0.011
Urinary microalbumin(U/L)	7.11(3.93,15.54)	10.02(3.80,19.68)	-1.262	0.207
Urinary transferrin(U/L)	0.21(0.1,0.46)	0.5(0.29,1.43)	-3.170	0.002
Urinary IgG(U/L)	2.14(0.85,3.7)	2(1,6.45)	-0.436	0.663
Urinary NAG(U/L)	12.77(9.00,20.265)	28.14(15.87,48.00)	-3.396	0.001

### Comparison of urinary kidney injury markers in the AKI and non-AKI groups

A total of 16 children were found to have AKI during hospitalisation, of which 11 were admitted to the paediatric ICU and the remaining 5 to the general paediatric medicine department. The AKI group had higher urinary NAG, α1-microglobulin, β2-microglobulin, urinary microalbumin, urinary transferrin and retinol-binding protein levels than the non-AKI group (*P* < 0.05) (see Table 3).

**Table 3:** Comparison of clinical data and urinary kidney injury markers the AKI and non-AKI groups

	AKI group (*n* = 16)	non-AKI group (*n* = 79)	*P value*
Gender			0.584
Male	7(43.8%)	43(54.4%)	
Female	9(56.2%)	36(45.6%)	
Age(y)	7.19 ± 3.13	6.63 ± 2.77	0.471
WBC (× 10^9^/L)	9.42 ± 3.92	9.49 ± 4.73	0.952
NE%	80.92 ± 12.55	65.65 ± 15.78	** < 0.001**
PLT (× 10^9^/L)	325(194.75 ~ 346)	315(260 ~ 411)	0.296
CRP (mg/L)	50.91(27.42 ~ 126.64)	10.84(4.7 ~ 40.68)	**0.005**
IL-6 (pg/mL)	15.96(5.63 ~ 34.54)	20.6(8.54 ~ 44.85)	0.382
ALT(U/L)	37.5(24 ~ 136.5)	22(14 ~ 43)	0.062
AST(U/L)	50.5(33.5 ~ 75.75)	31(22 ~ 44)	**0.004**
LDH(U/L)	156.5(44 ~ 371.75)	238(69 ~ 351)	**0.278**
CK(U/L)	603(120.75 ~ 1589.75)	139(40 ~ 302)	**0.006**
D-Dimer(ug/mL)	4.15(1.475 ~ 10.575)	1.8(1 ~ 4.1)	0.075
Serum IgG	7.95(5.925 ~ 9.375)	8.7(6.9 ~ 10.4)	**0.203**
Serum IgM	1.09(0.9425 ~ 1.955)	1.65(1.22 ~ 3.21)	**0.020**
GFR(ml/min/1.73m^2^)	71.83(68.29 ~ 75.37)	122.04(117.93 ~ 126.15)	** < 0.001**
Serum creatinine(umol/L)	71.16(62.66 ~ 79.65)	36.93(34.87 ~ 38.99)	** < 0.001**
Urine output(ml/24h)	315.63(230.19 ~ 401.06)	780.51(742.97 ~ 818.04)	** < 0.001**
α 1 microglobular protein(U/L)	13.8(5.525 ~ 27.85)	6.7(3.3 ~ 12)	** < 0.001**
β2-microglobulin(U/L)	0.92(0.24 ~ 2.715)	0.31(0.17 ~ 0.6)	**0.033**
Retinol binding protein(U/L)	0.65(0.18 ~ 4.35)	0.19(0.09 ~ 0.4)	**0.011**
Urinary microalbumin(U/L)	14.79(8.75 ~ 28.14)	7.48(3.66 ~ 16.56)	**0.004**
Urinary transferrin(U/L)	1.24(0.4 ~ 2.575)	0.3(0.1 ~ 0.6)	**0.001**
Urinary IgG(U/L)	2.25(1.425 ~ 15.625)	2(0.9 ~ 4.1)	0.214
Urinary NAG(U/L)	50.14(34.91 ~ 60.05)	16.47(9.14 ~ 28.23)	** < 0.001**

*WBC* White Blood Cell Count, *NE%* Neutrophil Percentage, *PLT* Platelet Count, *CRP* C-Reactive Protein, *IL-6* Interleukin-6, *ALT* Alanine Aminotransferase, *LDH* Lactate Dehydrogenase, *CK* Creatine Kinase

### Univariate logistic regression analysis

**Fig. 2:**
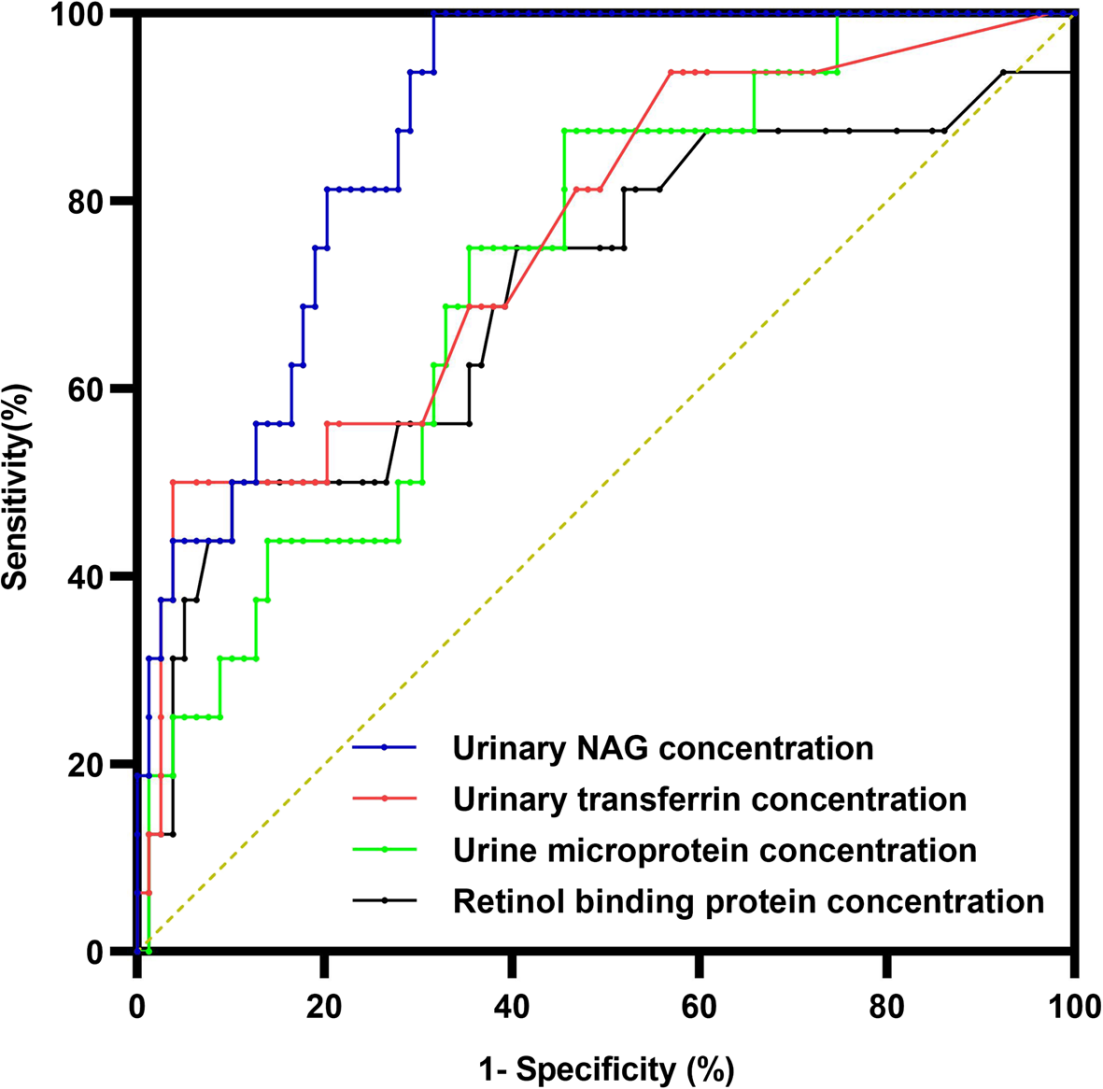
Analysis of the risk factors associated with AKI

A univariate logistic regression analysis was used to explore the early risk factors for AKI. The results indicated that the occurrence of AKI was related to the duration of hospitalisation and to the presence of pleural effusion, α1-microglobulin, urine microalbumin, urine transferrin, urine NAG, the neutrophil ratio and the levels of haemoglobin, CRP and serum IgM (*P* < 0.05), as shown in Table 4. The diagnostic value of the urinary kidney injury markers for AKI was analysed using the ROC curve (Table 5 and Fig. 2). The area under the curve in this study was between 0.7 and 0.9, and the test accuracy was high. This indicates that urinary NAG, urinary transferrin, urinary microalbumin and retinol-binding protein are highly accurate diagnostic tests for MPP-related AKI.

**Table 4:** Univariate logistic multivariate regression analysis for AKI

variable	b value	b standard error	Wald Chi-square value	*P* value	*OR* value
Duration of hospitalisation	0.082	0.034	5.807	0.016	1.086
Second-line antibiotics	1.680	0.607	7.662	0.006	5.367
Ventilator	3.483	0.710	24.051	0.000	32.560
Presence of pleural effusion	2.179	0.684	10.150	0.001	8.833
Times of bronchoscopy	0.571	0.175	10.604	0.001	1.769
α1 microglobulin	0.062	0.024	6.829	0.009	1.064
β2microglobulin	0.125	0.105	1.422	0.233	1.133
Retinol conjugated protein	0.097	0.065	2.217	0.137	1.102
Urine microalbumin	0.046	0.019	5.979	0.014	1.047
Urine transferrin	0.788	0.278	8.002	0.005	2.198
Urine IgG	0.052	0.029	3.202	0.074	1.503
Urine NAG	0.075	0.018	17.713	0.000	1.078
NE%	0.081	0.026	9.662	0.002	1.084
LY%	-0.088	0.030	8.743	0.003	0.916
Hb	-0.048	0.023	4.410	0.036	0.953
CRP	0.014	0.005	8.347	0.004	1.014
ERS	0.032	0.015	4.828	0.028	1.033
CK	0.001	0.000	8.231	0.004	1.001
Serum IgM	0.0693	0.336	4.249	0.039	0.500
FDP	0.066	0.026	6.431	0.011	1.068

**Table 5:** Diagnostic value of urinary kidney injury markers and urinary NAG in AKI

	AUC	95% CI	Cut-off	Sensitivity (%)	Specificity (%)
α 1 microglobular protein(U/L)	0.670	0.513 ~ 0.827	14.50	50	85
β2-microglobulin(U/L)	0.670	0.507 ~ 0.833	1.79	50	90
Retinol binding protein(U/L)	0.703	0.544 ~ 0.862	0.10	50	90
Urinary microalbumin(U/L)	0.729	0.604 ~ 0.854	17.35	88	54
Urinary transferrin(U/L)	0.761	0.628 ~ 0.894	0.1	50	96
Urinary IgG(U/L)	0.599	0.420 ~ 0.778	0.9	44	92
Urinary NAG(U/L)	0.879	0.807 ~ 0.951	10.55	100	68

### Multivariate logistic regression analysis and the establishment of the prediction model

**Fig. 3:**
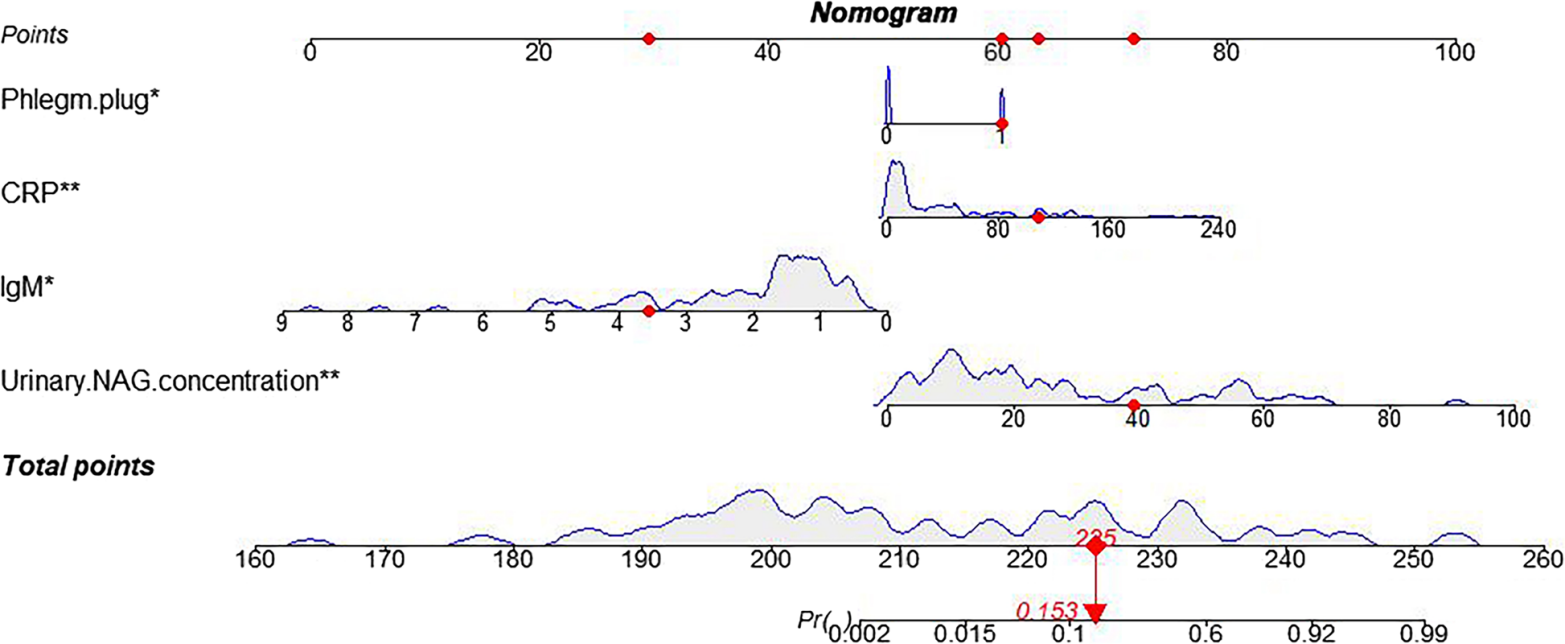
Multivariate Logistic regression analysis and the establishment of the prediction model. Note: The gray density map describes the distribution of various predictive factors and total scores among children with MPP included in the study. *: *P* ≤ 0.05, **: *P* ≤ 0.01; (The top horizontal axis represents the score scale. When the four variables take different values, they will correspond to different scores. Finally, the sum of the scores is obtained. The score according to the sum will be corresponding to a risk profile)

The variables with statistical significance in the aforementioned univariate analysis were included in a logistic multiple regression analysis, and the variables were screened using stepwise regression (Table 6). The results of the multivariate regression were used to select variables for the prediction model for MPP-related AKI in children. The final MPP-related AKI prediction model comprised four indicators: serum IgM, CRP, urinary NAG and sputum plug presence. The model nomogram of MPP-related AKI was constructed using these four indicators, as shown in Fig. 3.

**Table 6:** Multivariate logistic multivariate regression analysis for AKI

variable	b value	b standard error	Wald Chi-square value	*P* value	*OR* value
Serum IgM	-1.704	0.685	6.184	0.013	0.182
CRP	0.032	0.013	6.613	0.010	1.033
Urinary NAG	0.154	0.050	9.576	0.002	1.167
Sputum plug	2.156	1.059	4.144	0.042	8.639
Constant	-8.989	2.849	9.953	0.002	0.000

## Discussion

*Mycoplasma pneumoniae* pneumonia-related extrapulmonary complications are not uncommon. Although MPP-related skin and gastrointestinal manifestations, as well as cardiovascular system involvement, are relatively common extrapulmonary complications, MPP-related renal injury is relatively rare and has been the focus of relatively little research [6–8]. Acute kidney injury presents as a sudden and often reversible decrease in kidney function and can occur in patients with or without a previous history of kidney disease [9]. In China, the misdiagnosis rate of AKI in children is relatively high [10]. Although the symptoms of most children with AKI are mild and the prognosis is favourable, for some younger children or those with severe symptoms, the mortality rate is increased [11]. In terms of aetiology, sepsis is the primary risk factor for community-acquired AKI in preschool children and adolescents, and community-acquired pneumonia caused by *Mycoplasma pneumoniae* infection is also highly prevalent in this age group [12, 13].

This study found that various markers for urinary kidney damage can be abnormally increased to varying degrees in children with MPP; the most likely marker to increase is urinary NAG (68.42%). In the present study, the urinary NAG levels in the SMPP group were significantly higher than those in the non-SMPP group, and the AKI group had higher urinary NAG, α1-microglobulin, β2-microglobulin, urinary microalbumin, urinary transferrin and retinol-binding protein levels than the non-AKI group, with NAG levels being the most likely marker to be elevated. The MPP-related AKI prediction model was therefore determined to comprise four indicators: serum IgM, CRP, urinary NAG and sputum plug presence or absence. A nomogram of the MPP-related AKI prediction model was constructed.

A sputum plug is a specific manifestation of plastic bronchitis (PB) and can be identified through bronchoscopy. It is a phenomenon in which thick sputum forms blockages in the respiratory tract. In recent years, *Mycoplasma pneumoniae* is the most likely pathogen to cause PB [14]. Studies have shown that the formation of phlegm plugs may lead to insufficient pulmonary oxygenation. Long-term relative hypoxia causes an imbalance in the body’s oxygen supply and demand, thereby causing a strong systemic inflammatory response and immune dysfunction, leading to extrapulmonary complications such as AKI. The probability of complications is high [15].

The acute response protein CRP can be used as an indicator to evaluate the degree of inflammatory response and infection. The latest research shows that a significant increase in CRP can be highly suggestive of SMPP [16]. In addition, CRP is considered a factor that promotes the occurrence and progression of AKI by preventing the repair and proliferation of damaged renal tubular epithelial cells, increasing the inflammatory response and promoting the fibrosis of damaged renal tissue [17]. Some researchers believe that the excessive elevation of CRP may activate the coagulation system, constrict renal blood vessels and promote both inflammatory reactions and the formation of thrombus, thereby reducing renal blood flow and oxygen flowing through the kidneys, ultimately leading to the occurrence of AKI [18].

Serum IgM is an immunoglobulin that plays a key role in the body’s immune response. In the presence of septic shock and an inflammatory storm, the body’s immune function can deteriorate sharply in a short time, and the levels of immunoglobulins and complement proteins drop sharply [19]. Lobo et al. injected IgM into experimental mice and constructed a model of renal ischaemia, with the results revealing that compared with a control group, the incidence of AKI in the experimental group of mice was significantly reduced [20]. The nomogram in the present study also revealed that the higher the IgM level is, the lower the predicted risk of AKI.

Among the seven urinary kidney injury markers included in this study, increases in urinary NAG, β2-microglobulin, α1-microglobulin and retinol-binding protein suggest a certain degree of renal tubular damage, whereas urinary IgG and urinary transferrin indicate a certain degree of renal glomerulus damage. Increases in protein and urinary microalbumin are more likely to reflect glomerular damage [21]. The results of this study indicate that the probability of an increase in biomarkers related to renal tubular damage is greater than that for biomarkers related to glomerular damage.

Urinary NAG is the marker most likely to be elevated in children with MPP. It exists in the lysosomes of renal proximal tubule epithelial cells and has a molecular weight of 13–14 kD. When the proximal tubule cells are damaged, NAG is secreted into the urine. In recent years, the focus of research on urinary NAG has been its prediction of diabetic renal damage and early detection of renal transplant rejection. However, no research has focused on the relationship between urinary NAG changes and the severity of MPP in children or whether it can predict the occurrence of MPP-related kidney damage [22, 23]. The present study determined that urinary NAG could be used as a key indicator to predict the occurrence of MPP-related AKI. All the children in the study had mild AKI. We speculate that this is because kidney damage occurred at an early stage. The markers reflecting glomerular damage did not increase significantly. However, levels of urinary NAG were high, which further verifies the rationality of urinary NAG as an indicator of renal tubular damage.

Based on these findings, we speculate that the following mechanisms are the primary causes of the MPP and kidney damage: antigenic components similar to MP contained in various organs and tissues in the human body, or changes in the host cell membrane antigen structure caused by the MPP infection, which lead to the production of autoantibodies and trigger pathological immune reactions, resulting in lung and multi-organ damage [24]. Children with MPP-associated kidney damage exhibit cellular and humoral immune disfunction. Renal injury is a common immunopathological outcome in MPP, but the exact pathogenic mechanism remains unclear. In terms of the immune mechanism, the continuous positivity of MPP-IgM and IgG and transient or sustained decreases in complement C3 suggest that damage from circulating immune complexes to the kidneys may be a primary cause of kidney injury in affected children. Partially shared antigens exist between MPP antigens and glomeruli. Antibodies produced after infection form in situ immune complexes with glomerular self-antigens, leading to kidney damage. Alternatively, because MPP toxins damage the kidneys, some hidden antigens in the kidneys are exposed or new antigens generated, triggering autoimmune reactions and damage from circulating immune complexes to the kidneys, which are the main pathogenic mechanisms of post-MPP nephritis.

This study has some limitations. As this was a single-centre study with a small sample size, some bias may exist in the selection of enrolled cases and disease severity; therefore, data with a larger sample size may be needed to reduce the degree of bias. In addition, since sputum plugs can only be determined under bronchoscopy, primary hospitals that do not perform bronchoscopy may not be able to accurately determine the condition, preventing them from using the nomogram in this study to predict AKI. Another study also produced numerous nomograms on MPP-related phlegm plugs; primary doctors can therefore make decisions by combining the nomograms of phlegm plugs with the nomograms in this study [25]. This research team will continue to hone the study findings in future studies by increasing the sample size and formulating stricter and more standardised enrolment conditions and disease assumptions to develop a clinical prediction model appropriate for use by grassroots doctors.

## Conclusion

Urinary kidney injury markers are often elevated to varying degrees in children with MPP. Urinary NAG is the marker most likely to increase; this increase is particularly evident in severe cases. The prediction model nomogram, using IgM, CRP and urinary NAG levels as well as the presence or absence of sputum plugs as variables, can predict the probability of AKI in children with MPP.

### Supplementary Information

Supplementary Material 1.

## Data Availability

All data generated or analyzed during this study are included in this published article.

## Abbreviations

MPP: *Mycoplasma pneumoniae* Pneumonia
AKI: Acute kidney injury
SMPP: Severe MPP
NAG: N-acetyl-β-D-glucosaminidase
IgM: Immunoglobulin M
CRP: C-reactive protein
RMPP: Refractory MPP
ICUs: Intensive care units
ROC: Receiver operating characteristic
PB: Plastic bronchitis
