# Supplementary material for: Changes in urinary renal injury markers in children with Mycoplasma pneumoniae pneumonia and a prediction model for related early renal injury
Source: Ital J Pediatr. 2024 Aug 23;50:155. doi: 10.1186/s13052-024-01709-7 (PMC11342508; doi:10.1186/s13052-024-01709-7)
Supplement: Supplementary file 1 — Supplementary Material 1. [file 13052_2024_1709_MOESM1_ESM.docx]

**Supplementary Table1:** Comparison of abnormal elevations of urinary kidney injury markers between non-SMPP and SMPP groups

|  | **Reference Range (U/L)** | Non-SMPP group(n=41) | SMPP group(n=54) | χ^2^ value | *P* value |
| --- | --- | --- | --- | --- | --- |
| α 1 microglobular protein | 0-12.5 | 8(19.51%) | 19(35.19%) | 2.814 | 0.093 |
| β2-microglobulin | 0-0.3 | 20(48.78%) | 31(57.41%) | 0.698 | 0.404 |
| retinol binding protein | 0-0.7 | 4(9.76%) | 17(31.48%) | 6.388 | 0.011 |
| urinary microalbumin | 0-45 | 1(2.44%) | 2(3.7%) | 0.509 | 0.475 |
| urinary transferrin | 0-2 | 1(2.44%) | 8(14.81%) | 4.162 | 0.041 |
| urinary IgG | 0-10 | 3(7.32%) | 9(16.67%) | 1.845 | 0.174 |
| urinary NAG | 0.3-12 | 23(56.10%) | 42(77.78%) | 5.069 | 0.024 |
